# A Neonatal Resuscitation Curriculum in Malawi, Africa: Did It Change In-Hospital Mortality?

**DOI:** 10.1155/2012/408689

**Published:** 2011-11-20

**Authors:** Michael K. Hole, Keely Olmsted, Athanase Kiromera, Lisa Chamberlain

**Affiliations:** ^1^Stanford University School of Medicine, 251 Campus Drive, X215, Stanford, CA 94305, USA; ^2^Sutter Medical Center, Santa Clara Valley Medical Center, 751 South Bascom Avenue, San Jose, CA 95128, USA; ^3^St. Gabriel's Hospital, Namitete, Malawi

## Abstract

*Objective*. The WHO estimates that 99% of the 3.8 million neonatal deaths occur in developing countries. Neonatal resuscitation training was implemented in Namitete, Malawi. The study's objective was to evaluate the training's impact on hospital staff and neonatal mortality rates. *Study Design*. Pre-/postcurricular surveys of trainee attitude, knowledge, and skills were analyzed. An observational, longitudinal study of secondary data assessed neonatal mortality. *Result*. All trainees' (*n* = 18) outcomes improved, (*P* = 0.02). Neonatal mortality did not change. There were 3449 births preintervention, 3515 postintervention. Neonatal mortality was 20.9 deaths per 1000 live births preintervention and 21.9/1000 postintervention, (*P* = 0.86). *Conclusion*. Short-term pre-/postintervention evaluations frequently reveal positive results, as ours did. Short-term pre- and postintervention evaluations should be interpreted cautiously. Whenever possible, clinical outcomes such as in-hospital mortality should be additionally assessed. More rigorous evaluation strategies should be applied to training programs requiring longitudinal relationships with international community partners.

## 1. Introduction

There are 3.82 million neonatal deaths each year with a global neonatal mortality rate of 30 per 1000 live births [1]. The World Health Organization (WHO) estimates that each year 99% of those neonatal deaths occur in developing countries [2]. Neonatal mortality from birth asphyxia ranges from 40 to 610 neonatal deaths per 1000 live births [3–5], and nearly 1 out of every 4 neonatal deaths in Malawi is a result of birth asphyxia [6]. A lack of protocol and systemized training in neonatal resuscitation to reduce neonatal mortality secondary to birth asphyxia is common across sub-Saharan Africa [7]. As such, the United Nations' Millennium Development Goal 4—to reduce the 1990 mortality rate among under-five children by two-thirds by 2015—cannot be realized without educational efforts in neonatal resuscitation. As neonatal deaths make up 41% of under-5 mortality [8], neonatal resuscitation training is key.

Effective neonatal resuscitation is possible with basic equipment and skills in low-resource settings [9]. Case studies from China and India reveal that 90% of newborns with asphyxia require only drying, warming, and stimulation for complete revival [10, 11]. Such techniques coupled with ensuring a patent airway, suctioning, ventilation, administering oxygen, and chest compressions are part of the Neonatal Resuscitation Program (NRP) [12], an educational intervention based on the consensus of science and resuscitation guidelines of the International Liaison Committee on Resuscitation and published by the American Academy of Pediatrics and the American Heart Association. NRP is the developed world's standard of care to prevent death and complications of cerebral palsy due to asphyxia [13], and, when systematically implemented by trained personnel, has the potential to annually prevent 192 000 intrapartum-related neonatal deaths worldwide and 5–10% of deaths related to preterm complications [14]. Curriculums with components of NRP have significantly improved healthcare providers' knowledge, skills, and attitudes in developing countries [15, 16] and decreased neonatal mortality in the developing world up to 65.7 percent [10, 17, 18]. One study revealed declines in national trends of neonatal mortality over an 8-year period following NRP integration across Malaysia [19]. Furthermore, a 44.6% decline in neonatal mortality was seen with WHO's Essential Newborn Care (ENC) curriculum in Zambia, a resuscitation curriculum separate from the NRP [20]. Conversely, a recent study of ENC among 57 643 infants and NRP among 62 366 infants showed no reduction in mortality rates for either program [21]. Resource-constricted environments, often overflowing with competing needs and challenges like staff turnover, outdated equipment, and low levels of education, can render true change challenging to accomplish. Because the literature reports conflicting results following NRP training, a meta-analysis was performed which was inconclusive on all-cause neonatal mortality [22]. 

An increasing number of medical students and residents spend professional time working abroad [23, 24]. International health experiences have been shown to increase students' interest in public health, likelihood of choosing a career in primary care, and commitment to serving the underserved [25]. Many medical schools and residency programs now offer global health curriculum tracks, elective clinical rotations, and opportunities for research in developing world settings. Unfortunately, such research tends to examine short-term programs using pre- versus postintervention evaluations of participants while failing to evaluate patient outcomes. This reality is reflected in the current literature surrounding the assessment of neonatal resuscitation training efforts in the developing world [26–29].

Pediatric residents at Stanford University's Lucile Packard Children's Hospital (LPCH) developed a partnership with St. Gabriel's Hospital in Namitete, Malawi, Africa in January 2008. Stanford residents conducted a needs assessment in March 2008 that identified an interest in neonatal resuscitation training for the hospital's staff to reduce neonatal mortality. In response, Stanford residents developed and implemented NRP-based training to train St. Gabriel's Hospital physicians, clinical officers, and midwives (*n* = 14). The goal of this project was to evaluate the intervention on two levels: (1) the impact on trainees through a pre-/postcurricular evaluation and (2) the impact on neonatal mortality rates at St. Gabriel's Hospital.

## 2. Methods

### 2.1. Neonatal Resuscitation Training 

#### 2.1.1. Intervention

The curriculum was based upon the American Academy of Pediatrics NRP [12] and tailored in response to the constricted time and resources deemed feasible by on-site community leaders. The curriculum required a total of 6 hours: two hours of lecture, one hour of demonstration, and three hours of hands-on, scenario-driven sessions using mannequins to address components of NRP techniques, and oxygen was available (Table 1). During September of 2008, weekly training sessions for small groups of employees (*n* = 4-5) were held. The course was taught on one day by two Pediatrics residents in the same room with all 14 participants present.

#### 2.1.2. Evaluation

A survey tool was developed to assess pre-/posteducational intervention impact. The survey consisted of 18 queries covering three domains: attitude, knowledge, and skills. This tool was developed with the assistance of an expert in survey design, pilot-tested, and modified accordingly. Students' *t*-test was used to compare mean score changes for the pre-/post-educational intervention surveys. The pre-/postintervention instrument can be obtained by contacting the lead author.

### 2.2. Neonatal Mortality 

#### 2.2.1. Design

An observational, longitudinal study, and secondary data analysis was performed by data abstraction from existing hospital administration data provided by St. Gabriel's Hospital. Total neonatal deaths was the primary outcome for this analysis. Retrospective data revealed 20.9 neonatal deaths per 1000 live births at St. Gabriel's Hospital. Assuming a 44.6% decline [10, 17, 18, 20] to 11.6 neonatal deaths per 1000 live births, a two-tail test with 80% statistical power and a 95% confidence interval required at least 2956 subjects in both arms.

#### 2.2.2. Data Source

Health Facility Surveillance Forms (HFSFs), collected since 1971, contained anonymous data transferred from admission, delivery, and female ward books by a St. Gabriel's Hospital Records Assistant. The HFSFs included total number of deliveries, neonatal and maternal complications, and live births reported for each calendar month. The neonatal complications recorded included 5-minute Apgar 5 or less/asphyxia, neonatal sepsis, born-before-arrival, premature baby, baby less than 2500 grams, malformation, and HIV-infected. Maternal complications included prolonged obstructed labor, antepartum hemorrhage, postpartum hemorrhage, severe preeclampsia, eclampsia, puerperal sepsis, ruptured uterus, retained placenta, ectopic pregnancy, abortion, and anemia. Data for stillbirths were incomplete and thus unable to be analyzed. Birth datasets from 2006 to 2008 at St. Gabriel's Hospital showed approximately 3000 live births during any 15-month period. Thus, datasets for 15 months (June 2007 to August 2008) before intervention and 15 months (October 2008 to December 2009) after intervention were analyzed. Datasets from September 2008, the month of neonatal resuscitation training, were excluded. 

#### 2.2.3. Statistical Analysis

Contingency tables of neonatal mortality and survival and a Fisher's Exact Test were calculated to compare the proportion of neonatal mortality prior to and following the LPCH NPR curriculum intervention. The evaluation was based on total number of deaths during the two 15-month periods. A *P* value of less than 0.05 was considered statistically significant. SPSS version 17.0 software was used. The Stanford University Institutional Review Board determined that this study does not meet the Human Research Protection Program's definitions of human subject research because we did not obtain or receive private, individually identifiable information.

## 3. Results

Fourteen of 26 birth attendants at St. Gabriel's received training at St. Gabriel's Hospital in September 2008. The trainees consisted of two physicians, eight clinical officers, and four midwives. No trainees had previous neonatal resuscitation training and all trainees worked in the labor ward, antenatal unit, and female ward from October 2008 to December 2009. One trainee was further trained as an instructor. The pre-/posttest comparison evaluating all aspects of the module found that training scores improved from an average of 38.6% to 64.4%: attitude scores improved from 45% to 76.3%, knowledge scores improved from 30.4% to 58.7%, and skills scores improved from 57.5% to 75.5%, (*P* = .02) (Table 2). 

A total of 6 694 neonates born at St. Gabriel's Hospital were studied. There were 3 449 births preintervention and 3 515 births postintervention (Table 3, see the appendix). The neonatal mortality rate across the study's first 15 months (before intervention) was 20.9 neonatal deaths per 1000 live births compared to 21.9 neonatal deaths per 1000 live births in the 15 months after intervention, a 0.10% difference, (*P* = 0.86) (Table 3). The five-minute Apgar score less than 5 fell from 131 preintervention to 66 postintervention (Table 3). Complete data on neonatal deaths (to 28 days) was not available; only in-hospital deaths are recorded. Maternal mortality was 4.1 maternal deaths per 1000 live births preintervention compared to 2.0 maternal deaths per 1000 live births postintervention, (*P* = 0.24) (Table 3).

## 4. Discussion

The results of our study reject the hypothesis that neonatal mortality rates are lower at St. Gabriel's Hospital following the LPCH neonatal resuscitation curriculum intervention, despite the positive results of the pre-/postintervention evaluation. This highlights the necessity for evaluating interventions at the level of the primary outcome—in this case, neonatal mortality—and not relying on intermediary results like trainee attitude, knowledge, and skills. While the neonatal mortality rates before the LPCH neonatal resuscitation curriculum (20.9 neonatal deaths per 1000 live births) and after the intervention (21.9 per 1000) were not statistically different, it is interesting to note that the neonatal complication of a five-minute Apgar score less than 5 was decreased postintervention. It is possible that the LPCH neonatal resuscitation training had a positive impact for a small group of health providers reflected by the reduction of the five-minute Apgar score less than 5 from 131 preintervention to 66 postintervention. However, it appears that the clinical impact was not sufficient to reduce overall in-hospital mortality.

There are four possible explanations for the discordant results between our pre-/postintervention evaluation data and our mortality data. First, the modification of NRP required for the program's effectiveness within this time- and resource-deprived setting may have resulted in the unchanged rates of neonatal mortality. The modifications may have rendered the curriculum too short. What may be efficacious in a controlled setting might not be feasible or effective in developing communities. Second, factors external to the curriculum may have overwhelmed any statistically significant impact. For example, there was staff turnover with seven of the 14 health workers who received the LPCH neonatal resuscitation training continuing to work at St. Gabriel's. This likely had a negative impact on the level of neonatal resuscitation at deliveries. This creates a compelling case for the necessity of longitudinal train-the-trainer programs for St. Gabriel's staff. Third, our curriculum may not have addressed crucial aspects in the care of neonates. For example, the WHO Essential Newborn Care program addressed topics like cleanliness, temperature management, infection prevention, skin-to-skin care, breastfeeding, care of the small infant, and care of common illnesses. A recent study shows this program's relative effectiveness in reducing neonatal mortality rates [30]. Fourth, the LPCH neonatal resuscitation curriculum may not incorporate enough adult learning theory. Simulation-based training can be an effective educational methodology to promote skill acquisition and performance enhancement in neonatal resuscitation providers [31]. The LPCH neonatal resuscitation curriculum may be overwhelmed by didactical instruction (representing three of the six total hours) compared to hands-on activity. We hope that a qualitative assessment of the LPCH neonatal resuscitation training at St. Gabriel's Hospital will allow our community partner to make recommendations for further curriculum modifications.

Our findings are consistent with a study in Zambia that reports significant improvement in healthcare providers' knowledge and skills following curriculum intervention despite a limited application of curriculum guidelines due to local conditions [16]. Although NRP training reduces neonatal mortality in controlled, nonrandomized studies in China, India, and Africa [10, 15, 32, 33], the literature surrounding NRP curriculums' impact is not consistent. Our results contribute to that literature. These findings may have important implications for the children of the developing world born without adequate neonatal resuscitation services because of the complexity and high program cost [13, 34]. However, if 90% of asphyxiated newborns require only drying, warming, and stimulation for survival, then an abbreviated NRP-based curriculum could improve outcomes in an affordable way [10]. 

There has been a general progression in the evidence behind neonatal resuscitation. The WHO published its abbreviated NRP called Basic Newborn Resuscitation (BNR) in 1997 [35], a potential outline for future programs aimed at reducing developing world neonatal mortality. The assessment of BNR's effect on outcomes has been limited. It is possible that, like the LPCH neonatal resuscitation curriculum, such abbreviated education programs may not improve outcomes and be too costly in time. Health care workers' time away from patient care while in workshops can be a difficult obstacle to overcome, particularly in the developing world. However, Helping Babies Breath (HBB) [36], an evidence-based educational program released in June 2010 by the American Academy of Pediatrics, was developed to improve and be compatible with existing neonatal resuscitation programs experiencing the common obstacles of low-resource settings. HBB trains birth attendants in only the essential skills of newborn resuscitation (assessment, temperature support, stimulation to breathe, and assisted ventilation as needed) with particular emphasis on the first minute after birth. Studies to assess HBB's impact on outcomes are underway.

 Our study has five important limitations to consider. First, its retrospective analysis of large aggregate data brings inherent restrictions. Data at the individual patient level could have revealed a statistically significant impact of the LPCH neonatal resuscitation curriculum on neonatal mortality for certain subpopulations of neonates born at St. Gabriel's such as stillbirths. A prospective study, for example, in India found that while neonatal mortality did not decrease following the implementation of neonatal resuscitation programs, asphyxia-related deaths did significantly decline [33]. We were limited in our ability to individually track cases because such data is not collected at St. Gabriel's Hospital; thus, we were unable to compare the two cohorts on variables such as maternal age, prenatal health, and maternal health. Efforts to collect this individual patient data pose a significant administrative burden to our community partner. Second, the nature of the busy labor wards at St. Gabriel's Hospital poses a significant challenge to collecting data on how many deliveries are covered by individuals who received LPCH neonatal resuscitation training and if the skills learned during training are applied in postintervention months by trainees. Third, our results may be limited in generality. This was a unique NRP-based curriculum in a specific developing community. However, curricula must be tailored to meet the needs and realities of their settings. Fourth, we may have experienced a glass-ceiling effect. The Malawian neonatal mortality rate is low as compared to much of the developing world as it falls below the 30 deaths per 1000 live births target of Millennium Development Goal number 4 [6]. Finally, we were unable to compare the pre- and postintervention maternal complications given that the data were collected in aggregate. It was impossible to determine whether the observations were independent of one another as one female might have given birth during both the pre- and postintervention periods. Analytical approaches were limited due to the two samples violating the assumption of independence.

## 5. Conclusion

Global health interventions require robust evaluation. Short-term pre- versus postintervention assessments frequently show positive results, as ours did. These results are only proximal measures, however, and do not evaluate many projects' goals such as reducing neonatal mortality. We believe the primary contribution of this study is that it creates a compelling case for applying rigorous evaluation strategies to international health education initiatives. Longitudinal relationships with community partners are required for thorough evaluation. Short-term commitments can be unhelpful, costly in time and money, or even harmful. We aspire to practice evidence-based medicine for the betterment of patient outcomes as physicians in the United States. We should hold ourselves to the same, high standards when providing care to the developing world. 

## Figures and Tables

**Table 1:** LPCH neonatal resuscitation program curriculum^1^.

Hour	Method of instruction	Session content
1	Lecture	(i) Neonatal resuscitation curriculum overview and need
		(ii) Fetal circulation and oxygenation
		(iii) Fetal complications after labor
		(iv) Signs of compromised infant
		(v) Supplies needed for neonatal resuscitation
		(vi) Determination of need for resuscitation
		(vii) Initial assessment
		(a) Term
		(b) Amniotic fluid
		(c) Respirations
		(d) Tone
		(viii) Initial steps
		(a) Warming technique
		(b) Drying technique
		(c) Infant positioning to open airway
		(d) Clear airway
		(e) Stimulation technique
		(ix) Infant vitals
		(a) Respirations
		(b) Heart Rate
		(c) Color
		(x) Advanced techniques
		(a) Positive pressure ventilation
		(b) Chest compressions
		(xi) NRP-based special considerations adapted to local context

2-3	Demonstration with trainee practice and instructor feedback^2^	(i) Warming technique
		(ii) Drying technique
		(iii) Infant positioning for open airway
		(iv) Suctioning techniques
		(a) Oral
		(b) Nasal
		(c) Deep
		(v) Stimulation technique
		(vi) Bag-valve-mask technique
		(vii) Chest compression technique

4	Scenarios^3^	(i) Unexpected depressed infant
		(ii) Maternal preterm delivery and hemorrhage
		(iii) Hypothermia
		(iv) Meconium
		(v) Depressed infant due to sedation

5-6	Lecture	(i) Review
		(ii) Question and Answer Session

^1^NRP has 7 lessons: (1) Principles of Resuscitation, (2) Initial Steps in Resuscitation, (3) Bag and Mask Ventilation, (4) Chest Compression, (5) Endotracheal Intubation, (6) Medications, and (7) Special Considerations. The LPCH NRP-based curriculum incorporates lessons (1), (2), (3), (4), and (7).

^2^Infant mannequins are used for demonstrations and trainee practice sessions.

^3^NRP scenarios are adapted to local context and read by course instructor. Trainees react to the scenario while practicing resuscitation skills on infant mannequins. Instructor provides feedback as necessary on sequential steps of proper neonatal resuscitation for each scenario.

**Table 2:** Changes in trainees' attitude, knowledge, and skills in neonatal resuscitation.

Domain	Mean preintervention score (percent correct)	Mean postintervention score^1^ (percent correct)
Attitude Example: *“How comfortable are you with neonatal resuscitation?” * Likert scale, 1–4, Not at all to very comfortable, scored on quartiles: 1 =* * 25%, 2 = 50%, 3 = 75%, 4 = 100%	45	76
Knowledge Example: “*Restoration of adequate ventilation usually will result in a (rapid) (gradual) (slow) improvement in heart rate.” * Dichotomous correct or incorrect answer scored.	30	59
Skills Example: “*Please demonstrate the proper method of positive-pressure* *ventilation.” * Dichotomous correct or incorrect answer scored.	58	76

^1^Combined Students' paired *t*-Test with two-tailed distribution, (*P* = .02).

**Table 3:** Birth outcomes at St. Gabriel's Hospital before and after LPCH neonatal resuscitation training.

	Preintervention	Postintervention
Total deliveries	3449	3515
Total neonatal deaths	72	77
Total maternal deaths	14	7
Neonatal mortality	20.876 per 1000	21.906 per 1000
Maternal mortality	4.059 per 1000	1.991 per 1000
5-minute Apgar score less than 5	131	66

Fisher's exact test indicates that the difference between pre- and postintervention percent neonatal mortality is not statistically significant, (*P* = 0.86). The difference between pre- and postintervention percent maternal mortality is not statistically significant, (*P* = 0.24).

**Table 4:** Pre- versus postintervention neonatal mortality rate.

Time	Live births	Total neonatal deaths	Total deliveries	Percent mortality
June 2007	241	1	242	0.004132
July 2007	208	6	214	0.028037
August 2007	279	8	287	0.027875
September 2007	285	7	292	0.023973
October 2007	267	4	271	0.014760
November 2007	175	1	176	0.005682
December 2007	198	3	201	0.014925
January 2008	208	9	217	0.041475
February 2008	172	3	175	0.017143
March 2008	203	4	207	0.019324
April 2008	200	3	203	0.014778
May 2008	205	7	212	0.033019
June 2008	217	6	223	0.026906
July 2008	245	6	251	0.023904
August 2008	274	4	278	0.014388

Total preintervention	3377	72	3449	0.020876

October 2008	280	6	286	0.020979
November 2008	217	1	218	0.004587
December 2008	259	6	265	0.022642
January 2009	193	4	197	0.020305
February 2009	207	1	208	0.004808
March 2009	224	2	226	0.008850
April 2009	177	2	179	0.011173
May 2009	213	4	217	0.018433
June 2009	227	10	237	0.042194
July 2009	263	9	272	0.033088
August 2009	260	15	275	0.054545
September 2009	266	4	270	0.014815
October 2009	191	4	195	0.020513
November 2009	227	9	236	0.038136
December 2009	234	0	234	0.000000

Total postintervention	3439	77	3515	0.021906

## Appendix

For more details, see Table 4.

## Abbreviations

BNR: Basic newborn resuscitation
ENC: Essential newborn care
HFSFs: Health facility surveillance forms
HBB: Helping babies breathe
LPCH: Lucile Packard Children's Hospital
NRP: Neonatal Resuscitation Program
WHO: World Health Organization.
